# Phylogenetic structure of specialization: A new approach that integrates partner availability and phylogenetic diversity to quantify biotic specialization in ecological networks

**DOI:** 10.1002/ece3.8649

**Published:** 2022-03-01

**Authors:** Carlos J. Pardo‐De la Hoz, Ian D. Medeiros, Jean P. Gibert, Pierre‐Luc Chagnon, Nicolas Magain, Jolanta Miadlikowska, François Lutzoni

**Affiliations:** ^1^ 3065 Department of Biology Duke University Durham North Carolina USA; ^2^ Département des Sciences Biologiques Université de Montréal Montréal Québec Canada; ^3^ Biologie de l’évolution et de la Conservation Université de Liège Liège Belgium

**Keywords:** community phylogenetics, host–parasite interaction, hummingbird pollination, lichen symbiosis, mutualism, seed dispersal

## Abstract

Biotic specialization holds information about the assembly, evolution, and stability of biological communities. Partner availabilities can play an important role in enabling species interactions, where uneven partner availabilities can bias estimates of biotic specialization when using phylogenetic diversity indices. It is therefore important to account for partner availability when characterizing biotic specialization using phylogenies. We developed an index, phylogenetic structure of specialization (PSS), that avoids bias from uneven partner availabilities by uncoupling the null models for interaction frequency and phylogenetic distance. We incorporate the deviation between observed and random interaction frequencies as weights into the calculation of partner phylogenetic α‐diversity. To calculate the PSS index, we then compare observed partner phylogenetic α‐diversity to a null distribution generated by randomizing phylogenetic distances among the same number of partners. PSS quantifies the phylogenetic structure (i.e., clustered, overdispersed, or random) of the partners of a focal species. We show with simulations that the PSS index is not correlated with network properties, which allows comparisons across multiple systems. We also implemented PSS on empirical networks of host–parasite, avian seed‐dispersal, lichenized fungi–cyanobacteria, and hummingbird pollination interactions. Across these systems, a large proportion of taxa interact with phylogenetically random partners according to PSS, sometimes to a larger extent than detected with an existing method that does not account for partner availability. We also found that many taxa interact with phylogenetically clustered partners, while taxa with overdispersed partners were rare. We argue that species with phylogenetically overdispersed partners have often been misinterpreted as generalists when they should be considered specialists. Our results highlight the important role of randomness in shaping interaction networks, even in highly intimate symbioses, and provide a much‐needed quantitative framework to assess the role that evolutionary history and symbiotic specialization play in shaping patterns of biodiversity. PSS is available as an R package at https://github.com/cjpardodelahoz/pss.

## Graphical abstract

We developed an approach to determine the phylogenetic structure of the partners that interact with a focal species while accounting for their availability. When applied in four empirical network datasets, we found that a large proportion of species interact with phylogenetically random partners.

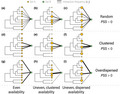

## 1 INTRODUCTION

Species interactions display patterns of biotic specialization that impact the evolution, assembly, and stability of biological communities (Chomicki et al., 2019; Guimarães et al., 2011; Poisot et al., 2011). These patterns result from a combination of trait‐driven and stochastic processes that enable interactions between organisms. For example, a pollination interaction between a hummingbird and a flowering plant may depend both on their morphological trait matching (e.g., flower corolla length and hummingbird bill length) and on the probability that the hummingbird will encounter the flower while foraging (Peralta et al., 2020; Sonne et al., 2020; Young et al., 2021). Multiple studies have quantified the relative importance of these two types of processes at the community level (Canard et al., 2014; Chávez‐González et al., 2020; Maglianesi et al., 2014; Simmons et al., 2019; Sonne et al., 2020; Stang et al., 2007; Vizentin‐Bugoni et al., 2014). As expected (Vázquez et al., 2009), traits that mediate species interactions often have predictive power for interactions and interaction frequencies (Maglianesi et al., 2014; Sonne et al., 2020; Vizentin‐Bugoni et al., 2014). However, in multiple cases, partner availability has been found to have a more important role in explaining species interactions (Canard et al., 2014; Chávez‐González et al., 2020; Simmons et al., 2019; Stang et al., 2007).

Typically, ecologists characterize biotic specialization by quantifying two properties of a species’ biotic niche: partner breadth and the intensity of interactions with those partners (Colwell & Futuyma, 1971; Futuyma & Moreno, 1988; Hurlbert, 1978; Pinheiro et al., 2016). Both of these properties can be shaped by partner availability. Therefore, the quantification of biotic specialization can be biased if the distribution of partner availabilities is not taken into account (Blüthgen et al., 2008). These biases emerge in multiple ways when studying specialization. For example, when species interactions are studied as networks, with nodes representing species and links representing interactions (Guimarães et al., 2006; Jordano, 1987), the total number of partners of a species, or node degree, characterizes partner breadth (Jordano et al., 2002), while the distribution of interaction frequencies across partners (interaction strength sensu Vázquez et al., 2007) provides information on the intensity of partner use. If interactions are random, a skewed distribution of partner availabilities will nevertheless result in rare species having high‐intensity interactions with a narrow set of partners (i.e., artifactual specialization on the most common species; Blüthgen et al., 2008).

Biotic specialization can also be studied using phylogenetic diversity metrics (Cooper et al., 2012; Doña et al., 2018; Esser et al., 2016; Lane et al., 2014). Here, species are considered more specialized if they associate with partners that are more closely related than expected by chance (Poulin et al., 2011). This approach acknowledges that simply counting partners may provide an incomplete picture of a species’ partner breadth. Even if two species associate with the same number of partners, one of them might be specialized on partners with a narrower range of traits (Dehling et al., 2020; Junker et al., 2013). However, many interactions are mediated by traits that are unknown or difficult to measure. Furthermore, most symbioses involve microbes for which species boundaries are unclear, making it difficult to quantify the number of partners (Magain, Miadlikowska, Goffinet, et al., 2017; Põlme et al., 2018; Toju et al., 2014). Because traits tend to be phylogenetically conserved (Goberna & Verdú, 2016; Swenson, 2013) and phylogenetic relatedness can be calculated without *a priori* species delimitation, phylogenetic diversity metrics are a useful alternative for characterizing the partner breadth of a species (Faith, 1992; Webb et al., 2002).

**FIGURE 1:**
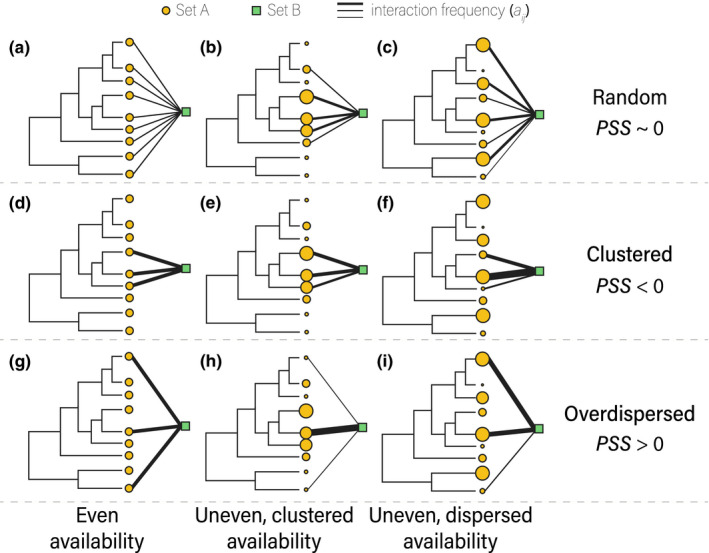
Schematic representation of three possible patterns of phylogenetic structure and their corresponding phylogenetic structure of specialization (PSS) values (random [a–c], clustered [d–f], and overdispersed [g–i]) across three different availability patterns of partners from set A that interact with one species from set B. The size of the orange circles represents the relative availability of each member of set A

Phylogenetic diversity metrics can also be biased by partner availability. For example, when interactions occur randomly, changes in partner availability can change interaction frequencies and alter the number of partners for a focal species (Figure 1a–c; Lessard et al., 2012; Poisot et al., 2015). As a result, a species may appear specialized on a set of closely related partners (i.e., phylogenetically clustered) only because the most available partners happened to be closely related (Figure 1b). Previous simulation studies have revealed multiple scenarios where failure to account for the distribution of species abundances (analogous to partner availability) results in biased estimates of phylogenetic diversity (Kembel, 2009; Miller et al., 2017). One way to account for this problem is to compare observed values of phylogenetic diversity to a null distribution generated by drawing partners from a pool of species in proportion to their availability, instead of drawing them with equal probabilities (Jorge et al., 2014; Kembel, 2009; Miller et al., 2017). However, this null model is also biased (Appendix S1; Figure S1) and results in an overestimation of non‐random phylogenetic structure with increasing interaction frequencies, as evidenced by Jorge et al. (2017) for networks of plant–herbivore interactions.

**FIGURE 2:**
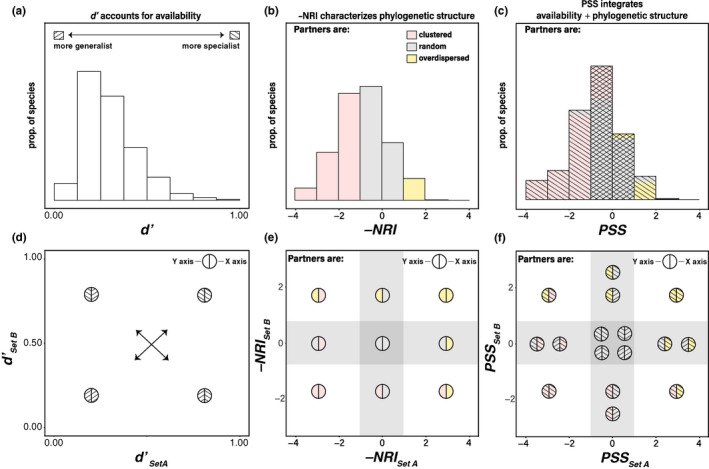
Schematic representation of the distribution of *d’* (a), −NRI (b), and PSS (c) values from a hypothetical community where most species associate opportunistically with their partners. Importantly, when using *d’*, the terms “specialist” and “generalist” refer exclusively to the interaction frequencies relative to the availability of the partners and completely ignore the actual number of partners (Blüthgen et al., 2006). For example, two species can have *d’* = 0 (generalists) even if one of them associates with 100 partners and the other species associates only with 10 partners, as long as both species associate with those partners in the same proportion as they are available. Panel c also shows how PSS values are related to *d’* and −NRI. Species with lower *d’* values will tend to have PSS values that are close to 0, while species with higher *d’* values can take values close to 0, but also positive and negative PSS values. Likewise, species with negative −NRI can take negative or near‐zero values with PSS, whereas species with positive −NRI can take positive or near‐zero values with PSS. Panels d, e, and f show a reference to interpret the distribution of species pairs in co‐specialization profiles of a bipartite interaction network involving set A interacting with set B, and vice versa. The X axis represents *d’*, −NRI, or PSS values for the species in the rows of an interaction matrix (set A), and the Y axis represents the *d’*, −NRI, or PSS values for the species in the columns of an interaction matrix (set B). The shaded gray areas in panels e and f show the −NRI and PSS space where clustering or overdispersion are not significantly different from a random pattern of phylogenetic structure (Figure 1a–c). These thresholds flanking the shaded gray areas (−1,1) represent the 95% confidence interval of the null distribution of −NRI or PSS simulated under each dataset. Each circle in panels d–f is labeled with the same legends as in panels a–c, with left semicircle corresponding to the value of the species in the Y axis (from the columns of the matrix), and the right semicircle representing the value of the species in X axis (from the rows of the matrix). For example, an interacting pair that falls in the lower left corner of the PSS space (f) involves two species that associate with phylogenetically clustered partners according to PSS, and likely have high value of *d’* (i.e., they are *d’* specialists)

The growing evidence that ecological interactions of many species are driven, at least at some scales, by the availability of potential partners (Canard et al., 2014; Chávez‐González et al., 2020; Simmons et al., 2019; Stang et al., 2007), calls for approaches to measure specialization that appropriately incorporate partner availability. We developed an index, phylogenetic structure of specialization (PSS), that integrates partner availability and phylogenetic diversity to measure biotic specialization in ecological networks. PSS avoids bias from uneven partner availabilities by uncoupling the null models for interaction frequency and phylogenetic distance. First, we quantify the deviation between observed interaction frequencies and random interactions. Next, we incorporate these deviations as weights into the calculation of partner phylogenetic α‐diversity; we also calculate phylogenetic α‐diversity for sets with the same number of partners but randomized phylogenetic distances among them. Finally, we compare observed and null values of the phylogenetic diversity metric to calculate the PSS index. Therefore, PSS is a measure of the phylogenetic structure of the partners of a focal species (partner breadth) that accounts for partner availability, helping to untangle trait‐driven and stochastic processes shaping patterns of specialization (Figure 1). We conducted simulations to detect potential biases of this new approach and found that PSS is not correlated with network properties as a previous index (Jorge et al., 2017; Appendix S1), which makes PSS comparable across datasets. We also illustrated the use of PSS with four empirical bipartite networks from the literature for which molecular phylogenetic trees were available for both sets of partners. We propose a conceptual framework to interpret phylogenetic structural patterns of biotic specialization in ecological networks (Figures 1 and 2) that enables the exploration of putative ecological and evolutionary processes generating these patterns.

## 2 METHODS

### 2.1 The phylogenetic structure of specialization index

Phylogenetic diversity indices used to measure specialization are standardized effect sizes (SES; Miller et al., 2017; Poulin et al., 2011). As such, these indices compare observed values of a phylogenetic diversity metric to a null distribution (i.e., SES = (null_mean_ − observed)/null_sd_). One strategy to account for partner availability is to generate the null distribution by calculating a phylogenetic diversity metric for sets of partners that are drawn from the pool of partner species in proportion to their availability (Jorge et al., 2014; Kembel, 2009; Miller et al., 2017). This approach uses a single null model for both interaction frequencies and phylogenetic distances. However, when a focal species associates with its partners nonrandomly, drawing from the pool of partner species in proportion to their availability will often yield a null set with a different number of partners than observed. This difference will grow larger as interactions become less random. If the null and observed values of the phylogenetic diversity metric are not based on the same number of partners, the phylogenetic diversity index (SES) will be biased (Figure S1 in Appendix S1; Jorge et al., 2017). This is because phylogenetic diversity metrics are not independent from the number of species upon which they are calculated.

We avoid this problem by uncoupling the null models for interaction frequency and phylogenetic distance. First, we use Kullback–Leibler distances (Kullback & Leibler, 1951) to quantify the magnitude of the deviation between the observed interaction frequencies and a null distribution representing random interactions (Blüthgen et al., 2006). We incorporate these deviations as weights into the calculation of the weighted mean pairwise phylogenetic distance, a common metric of phylogenetic α‐diversity (*wMPD*; Webb et al., 2008). Then, we calculate this modified *wMPD* for null sets of partners generated by shuffling taxa at the tips of the partner phylogeny, which randomizes the phylogenetic distances while keeping the number of partners constant. Finally, we compare the observed and null values of the phylogenetic diversity metric to calculate the PSS index as an SES. We describe the calculation of the PSS index below.

First, let **I** be an interaction matrix with *r* species in the rows (set A) and *c* species in the columns (set B). Each element *a_ij_* of **I** represents the interaction frequency between species *i* and species *j*, such that:

(1) $$I=\left[\begin{array}{ccc} a_{11} & \ldots & a_{1c}\\ \vdots & \ddots & \vdots \\ a_{r1} & \ldots & a_{\mathrm{rc}} \end{array}\right].$$

Let A*_i_* be the sum of interaction frequencies recorded for species *i*,

(2) $$A_{i}=\sum_{j=1}^{c}a_{\mathrm{ij}},$$

and let *m* be the sum of interaction frequencies across both rows and columns,

(3) $$m=\sum_{i=1}^{r}\sum_{j=1}^{c}a_{\mathrm{ij}}.$$

Let *q_j_* be a parameter expressing the relative availability of species *j*. We can define *q_j_* as the ratio of the sum of interaction frequencies of species *j* to the marginal sum of interaction frequencies in the matrix (i.e., matrix availability),

(4) $$q_{j}=\frac{A_{j}}{m}.$$

In this case, the availability parameter is inferred from the interaction matrix. Alternatively, *q_j_* can be determined empirically from measurements of partner abundance. The latter is preferable when such data are available (Jorge et al., 2014; Vizentin‐Bugoni et al., 2014). We will discuss the validity and implications of both approaches.

Let $P_{\mathrm{ij}}^{\prime }$ be the proportion of interactions of species *i* that are with species *j*,

(5) $$P_{\mathrm{ij}}^{\prime }=\frac{a_{\mathrm{ij}}}{A_{i}}.$$

We will use $P_{\mathrm{ij}}^{\prime }$ and *q* *_j_* to calculate the magnitude of the deviation of interaction frequencies from a null model where interaction frequencies are driven by partner availability. These magnitudes will be used below as weights in the calculation of the phylogenetic diversity metric. Before we calculate the weights, however, we will outline the calculation of the phylogenetic diversity metric, *wMPD*.

To calculate *wMPD*, we define M*_i_* as the set of *n* species that associate with species *i*, and **D** *_i_* as a symmetric matrix of pairwise phylogenetic distances between all species that belong to the set M*_i_*, such that:

(6) $$D_{i}=\left[\begin{array}{ccc} l_{11} & \ldots & l_{1n}\\ \vdots & \ddots & \vdots \\ l_{n1} & \ldots & l_{\mathrm{nn}} \end{array}\right].$$

Each element *l_ks_* of **D** *_i_* represents the phylogenetic distance between two partners of species *i*, species *k* and *s*. The weighted mean pairwise phylogenetic distance of M*_i_*, *wMPD_i_*, was defined by Webb et al. (2008) as:

(7) $$wMPD_{i}=\sum_{k\in M_{i}}\sum_{s\in M_{i}}\frac{l_{\mathrm{ks}}a_{\mathrm{ik}}a_{\mathrm{is}}}{\sum_{k\in M_{i}}\sum_{s\in M_{i}}a_{\mathrm{ik}}a_{\mathrm{is}}}.$$

This is a weighted mean of the pairwise phylogenetic distances among a set of species. It takes larger values for sets of distantly related species and smaller values for sets of closely related species. In this definition, the weight is the product of the interaction frequencies of species *i* with partners *k* and *s*, *a_ik_a_is_*. The *wMPD* is used to compute the SES_MPD_ index (Kembel, 2009), which is commonly used to quantify phylogenetic specialization (Cooper et al., 2012; Doña et al., 2018; Esser et al., 2016; Lane et al., 2014). SES_MPD_ is equivalent to −1 times the net relatedness index (−NRI; Webb et al., 2002), such that negative values correspond to clustering and positive values to overdispersion.

This metric can yield biased estimates of the phylogenetic structure, especially when the availability of the partners has phylogenetic signal (Kembel, 2009; Miller et al., 2017). To remove this effect, for a species *k* that associates with *i* and belongs to the set M*_i_*, we define a KL*_k_* factor as:

(8) $${\text{KL}}_{k}=P_{\mathrm{ik}}^{\prime }\text{ln}\left(\frac{P_{\mathrm{ik}}^{\prime }}{q_{k}}\right),$$

which expresses how much the interaction frequency of *i* with *k* deviates from a null model where *i* and *k* are interacting in proportion to their availability (i.e., randomly). This factor is an element of the sum used to compute Kullback–Leibler distances (Kullback & Leibler, 1951), which measure the difference between a probability distribution of interest and a reference distribution (i.e., null model). When interactions are random, the proportion of interactions of *i* with *k*, $P_{\mathrm{ik}}^{\prime }$, should converge to the availability of *k*, or *q_k_*. Therefore, the ratio between these two parameters tends toward 1, and KL*_k_* will approach 0. Conversely, when interactions are non‐random, $P_{\mathrm{ik}}^{\prime }$, is larger than *q_k_*, and KL*_k_* becomes larger than 0.

These distances are also used to calculate the species‐level specialization metric *d’* (Blüthgen et al., 2006), which sums equation 8 across all partners of species *i*. Here, we instead calculate a KL factor for each partner of species *i* and replace the interaction frequencies in equation 7, *a_ik_a_is_*, with KL factors for partner species *k* and *s*. This allows us to compute a version of the *wMPD* for species *i*, *klMPD_i_*, that is weighted by the KL factors instead of the interaction frequencies:

(9) $$klMPD_{i}=\sum_{k\in M_{i}}\sum_{s\in M_{i}}\frac{l_{\mathrm{ks}}{\text{KL}}_{k}{\text{KL}}_{s}}{\sum_{k\in M_{i}}\sum_{s\in M_{i}}{\text{KL}}_{k}{\text{KL}}_{s}},\text{KL}>0.$$

This mean of pairwise distances is now corrected for the availability of the partners through the KL weights. It takes larger values for species that interact non‐randomly with sets of more distantly related species, and it is undefined (0 in both numerator and denominator) for a species that interacts with its partners at the exact frequency that those partners are available (i.e., $P_{\mathrm{ik}}^{\prime }$= *q_k_* for every *k* that belongs to M*_i_*). However, the scenario where the index is undefined is extremely unlikely in natural networks and was never found in simulated networks. Equation 9 may result in negative values when KL factors are ≤ 0. Consequently, we only consider partners for which the KL factors are > 0. This means that we only use phylogenetic distances among partners that interact more frequently than expected under the null model where interaction frequencies are driven by availability. We assert in Appendix S2 that this is equivalent to excluding species from the partner set that have zero interactions with the focal species *i*, which does not affect the behavior of *klMPD*.

We can obtain a null distribution of *klMPD* values for the set of partners M*_i_* by randomly shuffling the tips of the partner phylogeny 999 times and calculating *klMPD* for each iteration. As we emphasized above, this maintains the observed total number of partners. Then, for each species *i*, we define PSS*_i_*,

(10) $${\text{PSS}}_{i}=\frac{klMPD_{i}‐mean\left(klMPD_{null_{i}}\right)}{sd\left(klMPD_{null_{i}}\right)},$$

as the difference between the observed value of *klMPD_i_* and the mean ${\mathrm{klMPD}}_{{\mathrm{null}}_{i}}$ divided by the standard deviation of the null values. PSS is thus an SES, with values close to 0 indicating that the partners of a focal species lack phylogenetic structure, and negative or positive values indicating phylogenetic clustering or overdispersion, respectively (Figure 1).

As a set‐level measure of the PSS, we take the mean of equation 10 from all species in the rows (set A) weighted by the interaction frequencies of each species:

(11) $${\text{PSS}}_{\mathrm{rows}}=\frac{1}{m}\sum_{i=1}^{r}{\text{PSS}}_{i}A_{i}.$$

Blüthgen’s *d’* (Blüthgen et al., 2006) and the −NRI index (Webb et al., 2002) measure specialization using availability and phylogenetic structure, respectively. PSS integrates both of these complementary sources of information to measure specialization (Figure 2a–c). For example, Figure 2a shows the distribution of Blüthgen’s *d’* values for the species in one set of a hypothetical bipartite network where most taxa associate opportunistically with their partners (i.e., most interactions are driven by partner availability), resulting in many species being generalists. However, *d’* does not provide information about the phylogenetic structure of the partners of those taxa. Conversely, the distribution of −NRI values for that same set of species (Figure 2b) indicates that a large fraction of the species interacts with phylogenetically clustered partners. However, since −NRI does not incorporate availability, some of the species may appear as interacting with clustered partners as a result of a biased distribution of partner availabilities (e.g., Figure 1b). If that were the case, the distribution of PSS values would show that the largest fraction of the species associates with phylogenetically random partners (i.e., be equal to or close to 0), since PSS integrates both availability and phylogenetic structure (Figure 2c).

For bipartite interaction networks, the distribution of values of the specialization indices can be visualized in two dimensions, where all pairs of interacting species are plotted according to the specificity values for each partner. For example, an interaction between two species with low *d’* values would occupy the generalist–generalist region (lower left corner) in Figure 2d. That same pair of species can occupy any area of the −NRI space in Figure 2e, because the −NRI values do not include partner availability in the estimation of phylogenetic structure. In contrast, the same pair of species can only occupy the random–random area of the PSS space (Figure 2d,f), because for PSS to yield a value that is significantly different from 0, a species must interact with its partners more than expected given their availability, and those partners must display a significant phylogenetic structure. Conversely, a pair of species with high *d*’ can occupy any region of the PSS space (Figure 2d,f), because the phylogenetic structure of their partners may be clustered, overdispersed, or random, even if they interact with those partners more than expected given their availability.

### 2.2 Why use the mean pairwise phylogenetic distance for PSS?

Metrics of phylogenetic α‐diversity fall into one of the three groups based on what they quantify (Swenson, 2014; Miller et al., 2017; but see Tucker et al., 2017 for an alternative classification): (i) mean relatedness among species, such as the mean pairwise phylogenetic distand (MPD; Webb, 2000); (ii) relatedness of species to their closest relatives, such as the mean nearest taxon distance (MNTD; Webb, 2000); or (iii) total tree length, such as Faith’s phylogenetic distance (PD; Faith, 1992). Our approach to account for availability could be coupled with any metric of phylogenetic α‐diversity, that is, by incorporating the KL factors as weights in the calculations of the mean. However, there are caveats associated with specific types of metrics. For example, metrics from group ii only provide insights about fine‐scale phylogenetic structure because only the closest relatives are considered. Additionally, the value of metrics from group iii increases monotonically with the number of partner species and, therefore, does not provide information about phylogenetic structure. For example, Faith’s PD may give the same value for two distantly related taxa as well as for five closely related taxa. The use of MPD for our PSS index allows the detection of three different phylogenetic structural patterns of specialization (random, clustered, and overdispersed; Figure 1).

However, MPD can only be calculated if one lineage is interacting with at least two partners. This is problematic in highly specialized symbiotic systems where many species interact with one partner. If we assume that a species with one partner has a MPD of 0, as in previous studies (Jorge et al., 2014, 2017), PSS cannot be calculated because the null distribution would also be estimated with one partner, resulting in an undefined PSS with the numerator and denominator of equation 10 being equal to 0. We solved this problem by assuming the existence of a sister taxon to the single partner of the focal species. This new sister taxon is joined to the original partner with a branch length that is half the minimum pairwise distance recorded between any pair of species in the phylogeny of the partners. The observed interaction frequencies of the original partner are equally divided between the sister taxa. This keeps the relationship between interaction frequencies and availability (i.e., P′_*ik*_/*q*_*k*_ in equation 8) constant; therefore, the new sister taxon has the same KL factor as the original taxon. This new sister taxon is only added for the calculation of *klMPD*_*i*_ (equation 10) for species with one partner and does not affect species that have more than one partner.

### 2.3 Testing the PSS index using simulations

#### 2.3.1 Varying dimensions and marginal sum of interaction frequencies

We simulated random matrices using the genweb() function in the R package *bipartite* (Dormann et al., 2008), which relies on three parameters: number of columns (N1), number of rows (N2), and average interaction frequency per link (dens). These values are used to calculate the sum of all interaction frequencies of the simulated matrix (*m*), as *m *= N1×N2×dens. Then, the simulated matrix of dimensions N1×N2 is populated with *m* interactions, such that the marginal sums of the interaction frequencies in the rows and the columns follow a lognormal distribution. This procedure results in heterogeneous distributions of both the number of links per species and the interaction frequencies per link.

We simulated three sets of random matrices. In the first set, we assessed the values of PSS on random symmetric matrices with dimensions varying from 5×5 to 200×200. The second set consisted of random matrices with unequal numbers of columns and rows varying from 2×20 to 200×20. The average interaction frequency per link (dens) was kept at 2 for all matrix configurations in the first two sets. The third set contained random matrices with increasing marginal sum of interaction frequencies (*m *= 5–4000) and fixed dimensions (50×50). We accomplished this by varying “dens” within the genweb() function in *bipartite*. The marginal sum of interaction frequencies (*m*) sets a limit to the number of binary links in the simulated matrices because the marginal totals are constrained to follow a lognormal distribution. Therefore, the random matrices in the third set also span a range of connectance values, with matrices with higher *m* having higher binary connectance (Figure S2). Each simulation step consisted of: (i) a random matrix simulated as described above and (ii) a random ultrametric tree, with the matrix columns as taxa generated with the function rcoal() in the R package *ape* (Paradis et al., 2004). We then calculated PSS*_rows_* for each matrix. Each simulation step was replicated 10 times. In addition, each set of simulations was performed three times, with branch length distributions of the random trees drawn from either a lognormal, normal, or uniform distribution. We estimated the rate of Type I error as the proportion of simulated matrices for each parameter value for which the observed *klMPD* value was significantly different (α = 0.05) from the null distribution generated as described for equation 10.

#### 2.3.2 Varying nestedness and modularity

To determine whether network structural patterns constrain the possible values of PSS, we tested for correlation between PSS and matrices simulated with varying degrees of nestedness and modularity. In nested networks, specialist species tend to interact with a subset of the partners associated with generalist species (e.g., Guimarães et al., 2006). In modular networks, groups of species share a set of preferred partners, resulting in compartmentalized networks (e.g., Chagnon et al., 2018; Olesen et al., 2007). In order to simulate matrices with a gradient of nestedness and modularity values, we started by creating a perfectly nested and a perfectly modular 50×50 binary matrix. Then, each simulation step swapped the positions of a 0 and 1 in the matrices, thus adding noise and decreasing nestedness and modularity (Chagnon, 2015). Although we used binary matrices, we treated them as quantitative so that the network structure could be manipulated in a predictable way. After each step of the nestedness simulation, we calculated nestedness using wNODF (weighted nestedness metric based on overlap and decreasing fill) developed by Almeida‐Neto et al. (2008) and implemented in *bipartite*. After each step of the modularity simulation, we calculated the modularity (Q) using the simulated annealing algorithm developed by Dormann and Strauss (2014) implemented in *bipartite*. For each simulation, we generated a random ultrametric tree with the matrix columns as taxa using the function rcoal() in *ape*. We then calculated PSS*_rows_* for the matrices of each simulation step. The availability of the partner species (*q_j_*) is calculated as if the network were quantitative (see equations (2), (3), (4)). Even if all interaction frequencies are 0 or 1, our simulation strategy still generates heterogeneity in the availability of partners (*q_j_*) and the KL weights (equation 8) that are used to calculate PSS. Each simulation step was replicated 20 times for both modularity and nestedness analyses. As above, each set of simulations was performed three times, with branch length distributions of the random trees drawn from either a lognormal, normal, or uniform distribution. Type I error rates were estimated as described above.

#### 2.3.3 Can PSS detect clustering and overdispersion?

There are no models to simulate phylogenetic networks with specific patterns of phylogenetic structure, and developing them is beyond the scope of this paper. However, a recent study developed a simulation framework to explore the statistical behavior of a comprehensive set of phylogenetic diversity metrics when applied to communities (Miller et al., 2017). Although we are applying PSS to interaction networks, PSS is an index of phylogenetic diversity and can be used to measure phylogenetic diversity in communities. This is because community data matrices (CDMs) are quantitatively analogous to interaction matrices. In a CDM, the rows correspond to spatial plots and the columns correspond to species. Likewise, in a CDM, the availability parameter is analogous to the regional availability of species, and it is important to account for its role in the sorting of species into plots of a CDM (Lessard et al., 2012; Miller et al., 2017). The important difference is that in a CDM, only the columns (species) have a phylogenetic tree. Therefore, we can only calculate PSS for each of the plots (rows) in a CDM.

To determine whether PSS can detect phylogenetic structure patterns (Figure 1) when they are present (Type II error rate), we used the approach developed by Miller et al. (2017). This allowed us to compare PSS to existing methods. This framework simulates arenas where individuals are spatially distributed according to their phylogenetic relatedness (Appendix S3). We then sampled the species composition of plots within these arenas to create CDMs. The PSS index was calculated for the plots (rows) within these CDMs. Rates of Type II error were calculated at the CDM and plot level as described in Appendix S3.

### 2.4 Empirical networks with phylogenies and with or without empirically estimated availability

We used four bipartite networks from the literature for which molecular phylogenetic trees were available for both sets of partners: (i) mammals–fleas, an antagonistic network of interactions between small mammals and their ectoparasitic fleas (order Siphonaptera) that were sampled in four regions of Slovakia (Stanko et al., 2002); (ii) avian seed‐dispersal, a mutualistic network of bird seed‐dispersal interactions compiled from studies conducted across multiple localities in the Brazilian Atlantic Forest (Bello et al., 2017); (iii) cyanolichens, a mutualistic network of interactions between species of the fungal lichen‐forming genus *Peltigera* and their cyanobacterial partners from the genus *Nostoc*, which were recorded at a global scale as part of phylogenetic studies on *Peltigera* (Lu et al., 2018; Magain, Miadlikowska, Goffinet, et al., 2017; Magain, Miadlikowska, Mueller, et al., 2017; Magain et al., 2018; Miadlikowska et al., 2014, 2018; O’Brien et al., 2005, 2013; Pardo‐De la Hoz et al., 2018) and compiled by Chagnon et al. (2019); and (iv) hummingbird pollination, a mutualistic network of pollination interactions between hummingbirds and plants in the Colombian Andes (network 7 in Sonne et al., 2020). Table 1 shows a summary of these four datasets. For each species in each of these datasets, we calculated node degree, the interaction frequency‐based specialization index *d’* (Blüthgen et al., 2006), the phylogenetic diversity index −NRI (Webb et al., 2002), and PSS. The availability parameter (*q_k_*; equation 8) was estimated from the interaction frequencies in the matrices for all datasets as indicated in equation 4. We also estimated PSS values using empirical abundance data that were available for the fleas in the mammals–fleas dataset (Table 1; Stanko et al., 2002) and for both sets of species in the hummingbird pollination dataset (Sonne et al., 2020).

**TABLE 1:** Summary of features of the four empirical datasets used in this study

Dataset	Marginal sum of interaction frequencies	Taxonomic scale	No. of taxa	Phylogenetic distances	Empirical availability	Phylogeny source
Mammals–fleas						
Mammals	11,509	Species	19	Substitutions per site	Yes	Bininda‐Emonds et al. (2007)
Fleas		Genus	14	Substitutions per site	No	Zhu et al. (2015)
Avian seed‐dispersal						
Birds	2474^a^	Species	183	Divergence times	No	Emer et al. (2019)
Plants		Species	270	Divergence times	No	Emer et al. (2019)
Cyanolichens						
Fungi	1026	Species	155	Substitutions per site	No	Chagnon et al. (2019)
Cyanobacteria		Phylogroups and haplotypes^b^	95	Substitutions per site^c^	No	Chagnon et al. (2019)
Hummingbird pollination						
Hummingbirds	3664	Species	14	Divergence times	Yes	McGuire et al. (2014)
Plants		Species	23	Divergence times	Yes	Zanne et al. (2014)

^a^ We limited our sampling to avian seed‐dispersal interactions that were recorded as part of network studies to limit sampling biases towards the plants or the birds present in the communities.

^b^ Both phylogroups and haplotypes were used as proxies for species (Magain, Miadlikowska, Goffinet, et al., 2017).

^c^ Sequence pairwise distances, corrected with the General Time Reversible model because we lacked well‐resolved and well‐supported phylogenies for *Nostoc* and, therefore, could not infer phylogenetic distances directly from branch lengths.

### 2.5 R package for computing PSS values

We developed an R package (https://github.com/cjpardodelahoz/pss) with functions to compute PSS using interaction matrices and phylogenetic trees from the interacting species as input. Our R package has function dependencies from the R packages *ape*, *bipartite*, *picante* and *vegan* (Dormann et al., 2008; Kembel et al., 2010; Oksanen et al., 2019; Paradis et al., 2004), and includes code modified from Swenson (2014).

## 3 RESULTS

### 3.1 PSS is independent from basic network features

We calculated PSS across simulated bipartite matrices lacking phylogenetic structure but varying in size, number of rows and columns, marginal sum of interaction frequencies, nestedness, and modularity. We found no correlation between any of these network structural variables and PSS values (Figure S3c), suggesting that our approach allows for comparisons across different systems with a wide range of network properties. Type I error rates were between 0% and 10% (mean 4%), except for small and equal numbers of rows and columns (< 11 rows × < 11 columns; Figure S4a), small network matrices with unequal numbers of rows and columns (< 15 rows × 20 columns; Figure S4b), and networks with low marginal sum of interaction frequencies (< 30 interactions; Figure S4c). This was expected because in these cases most species have a single interaction recorded, which means that their node degree is equal to 1. As a consequence of the strategy that we implemented to calculate PSS when a species has a single partner, these taxa appear specialized on a phylogenetically clustered lineage.

Rates of Type II error at the CDM level were low for both clustered (1.1%) and overdispersed (5.2%) scenarios. We observed high rates of Type II error (36%) in clustered scenarios when assessed at the plot level, which correspond to single rows in the CDMs.

### 3.2 Comparison of PSS and −NRI

**FIGURE 3:**
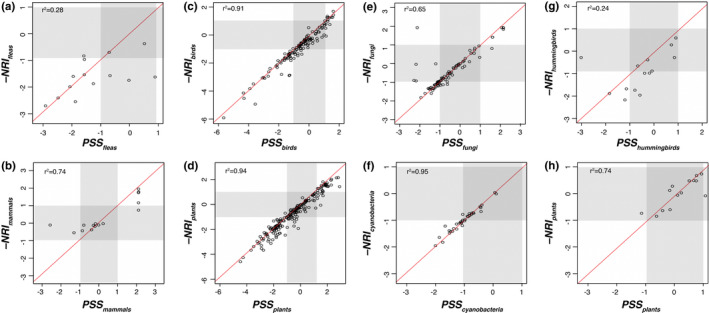
Comparison of −NRI and PSS values for the species in each of the eight sets present in the four empirical datasets we analyzed. If a circle falls along the red diagonal line, it means that the two metrics being compared yield the same value for that particular species. (a and b) fleas and their mammalian host, (c and d) birds as dispersers of plant seeds, (e and f) lichen‐forming fungi and their cyanobacterial partners, and (g and h) hummingbirds and the plants they pollinate. The shaded areas show the region of −NRI and PSS space where clustering or overdispersion is not significantly different from a random pattern of phylogenetic structure. The thresholds of the shaded areas were defined as the mean of the 95% confidence interval of the null distributions generated for each taxon in the datasets. All PSS values were calculated with the availability parameter estimated from interaction frequencies. See Figure 5 for a comparison of PSS values based on direct empirical estimations of availability versus interaction frequencies as a proxy for availability

PSS and −NRI generally yielded similar results regarding the proportion of taxa and interaction frequencies with random, clustered, and overdispersed partners (Table 2). However, these indices can lead to different results for some datasets. For example, in the avian seed dispersal dataset, 48% of plant taxa were found to associate with clustered partners according to PSS, compared to 32% according to −NRI (Table 2). In some cases, such as in the avian seed‐dispersal dataset, PSS and −NRI inferred a similar percentage of plant species that associate with random partners (39% and 37%, respectively). However, those species account for different percentages of the interaction frequencies (40% and 29%, respectively). This indicates that PSS and −NRI detected different species that have random partners. These discrepancies are more evident in the comparison of the index values obtained for each taxon (Figure 3). These indices yielded highly similar values for some sets (Figure 3c,d,f,h) and very different values for taxa in other sets (Figure 3a,b,e,g). In two cases, the correspondence between values of the two indices was much higher for one of the sets within the same dataset: fleas *r* ^2^ = .28 (Figure 3a) compared to mammals *r* ^2^ = .74 (Figure 3b); and hummingbirds *r* ^2^ = .24 (Figure 3g) compared to plants *r* ^2^ = .74 (Figure 3h). These are also the two cases where we have empirical data for the abundances rather than relying on matrix availabilities (Table 1).

**TABLE 2:** Comparison of PSS and −NRI values estimated for taxa across the four empirical datasets used in this study

	Random		Clustered		Overdispersed	
	PSS	−NRI	PSS	−NRI	PSS	−NRI
Mammals–fleas						
Mammals	43 / 94	63 / 95	29 / 1	0 / 0	29 / 6	21 / 5
Fleas	36 / 7	29 / 5	64 / 93	71 / 95	0 / 0	0 / 0
Avian seed‐dispersal						
Birds	42 / 44	40 / 43	50 / 44	29 / 50	8 / 12	6 / 5
Plants	39 / 40	37 / 29	48 / 47	32 / 58	14 / 13	12 / 11
Cyanolichens						
Fungi	31 / 38	31 / 44	63 / 49	24 / 25	6 / 12	5 / 11
Cyanobacteria	54 / 52	18 / 35	46 / 48	20 / 55	0 / 0	0 / 0
Hummingbird pollination						
Hummingbirds	71 / 63	57 / 55	29 / 37	43 / 45	0 /0	0 / 0
Plants	52 / 89	61 / 91	35 / 4	0 / 0	13 / 7	9 / 6

Note

−NRI cannot be calculated for taxa that associate with a single partner. Therefore, we were not able to calculate −NRI for 100% of taxa in some datasets. Values before backslash are percentage of taxa, and values after backslash are percentage of interaction frequencies. Totals within a species set and index may not sum to 100 due to rounding. All calculations were based on interaction frequencies as a proxy for availability. See Figure 5 for a comparison of PSS values based on direct empirical estimations of availability versus interaction frequencies as a proxy for availability.

**FIGURE 4:**
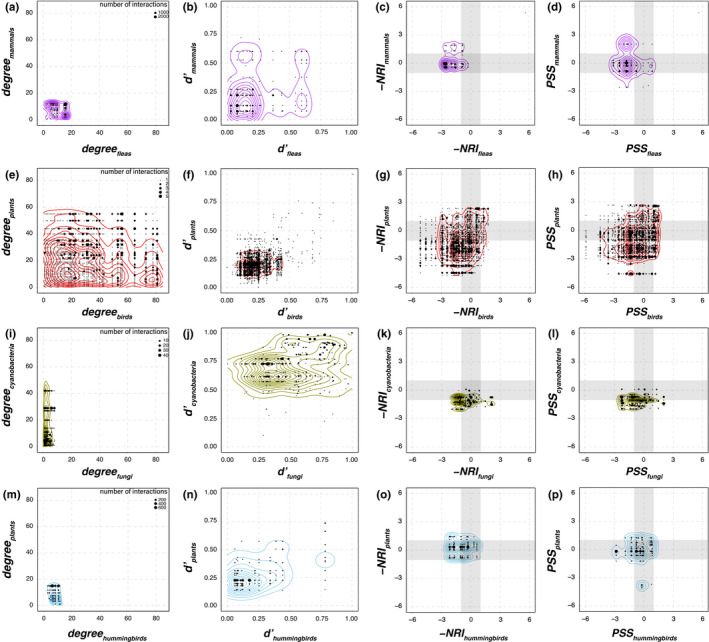
Comparison of biotic co‐specialization profiles of four empirical bipartite networks using four metrics (columns): node degree, Blüthgen’s *d’*, −NRI and PSS. (a–d) mammals–fleas network from Slovakia (Stanko et al., 2002). (e–h) avian seed‐dispersal network from the tropical Atlantic Forest (Bello et al., 2017). (i–l) cyanolichen network from an opportunistic global sampling of the lichen‐forming fungal genus *Peltigera* and their *Nostoc* cyanobacterial partners (Chagnon et al., 2019). (m–p) hummingbird pollination network from the Colombian Andes (Sonne et al., 2020). Each dot on these plots represents a pair of interacting species. These dots are placed on graphs according to the biotic specificity metric value of the interacting species. For example, in panel i, fungi are interacting with one to eight phylogroups of cyanobacteria (X axis) while cyanobacteria are interacting with one to more than 40 fungal species (Y axis) in this network of cyanolichens. All PSS values were calculated with the availability parameter estimated from interaction frequencies. See Figure 5 for a comparison of PSS values based on direct empirical estimations of availability versus interaction frequencies as a proxy for availability. The shaded areas in the panels of the third and fourth columns represent non‐significant clustering or overdispersion. The thresholds were defined as the mean of the 95% confidence interval of the null distributions generated for each taxon in the datasets. The size of the dots represents the number of times an interaction was recorded in the matrix, i.e., interaction frequency. Contour lines are estimated 2D distributions

Even in cases where the two indices inferred the same structure, we observed a slight trend towards more negative values for the −NRI index (Figure 3c,d). For example, most interaction pairs (dots) from the mammals–fleas dataset fall into the same areas (Figure 2e,f) of the −NRI (Figure 4c) and PSS spaces (Figure 4d). However, the interaction density is shifted towards the left (more negative) according to −NRI_fleas_ (Figure 4c) compared to PSS_fleas_ (Figure 4d). We found a similar result with the avian seed‐dispersal dataset, for which −NRI inferred a higher density of interactions in the clustered–clustered area (Figure 4g) compared to PSS (Figure 4h). In contrast, PSS inferred more negative values than −NRI for lichen‐forming fungi (Figures 3e and 4k,l).

### 3.3 Most taxa interact with phylogenetically random or clustered partners

**FIGURE 5:**
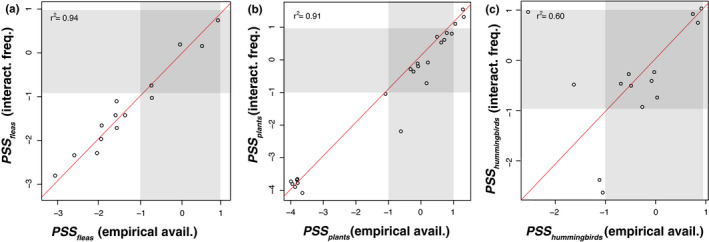
Comparison of PSS values estimated using empirical estimates of availability (empirical avail.) obtained from surveys of species abundances (X axis) vs using the marginal sum of interaction frequencies (interact. freq.) obtained from an interaction matrix as a proxy for availability (Y axis). PSS of (a) mammal partners of flea species, (b) plants in the hummingbird pollination dataset, and (c) the hummingbirds. Each circle on these plots represents a species. The thresholds for the shaded areas (random phylogenetic structure) were defined as the mean of the 95% confidence interval of the null distributions generated for each taxon in the datasets. Points on the diagonal red line indicate identical PSS values

Although we found a wide range of variation in the number of partner species (node degree) in the empirical networks (Figure 4a,e,i,m), most interaction pairs involved species without strong specialization signal according to Blüthgen’s *d’* (Figures 2d and 4b,f,n). The cyanolichen network was an exception, with multiple interaction pairs involving specialist cyanobacteria and generalist (opportunistic) lichenized fungi, or both specialist cyanobacteria and specialist fungi (Figure 4j). Across all four interaction networks, both −NRI and PSS indicated that many taxa interact with random and clustered partners (Table 2; Figure 4c,d,g,h,k,l,o,p). However, taxa that interact with overdispersed partners were rare and not found in all sets (Table 2; Figure 4c,d,g,h,k,l,o,p**)**. PSS values for the fleas in the mammals–fleas dataset and the plants in the hummingbird pollination dataset were highly similar when calculated based on empirically estimated availability versus interaction frequencies as a proxy for availability (Figure 5a,b). In contrast, empirical and matrix availabilities yielded different PSS values for multiple hummingbird species (Figure 5c).

## 4 DISCUSSION

### 4.1 PSS is informative and robust to error types

Phylogenetic structure of specialization integrates both partner availability and phylogenetic structure to characterize biotic specialization of species within interaction networks. As expected, when availability is roughly equal among partners (Figure 1a,d,g), PSS captures similar information in empirical networks as −NRI (Figure 3c,d,f,h), an index that accounts for phylogenetic structure without considering partner availability. Therefore, cases where the two indices diverge (Figure 3a,b,e,g) are likely due to unequal partner availabilities (e.g., Figure 1b,c). These are situations where existing approaches that do not account for partner availability, such as −NRI, can infer clustered or overdispersed phylogenetic structure when the phylogenetic pattern is actually random (i.e., Type I error), or may fail to detect clustering and overdispersion (i.e., Type II error; Kembel, 2009; Miller et al., 2017).

The rates of Type I and Type II error observed for PSS are comparable to the best performing combination of phylogenetic diversity metric +null model as reported in a previous study (*wMPD* + regional null; Miller et al., 2017). However, that combination is designed to describe communities with many species, which limits its application to interaction networks where species have few partners (Appendix S1). Furthermore, PSS values are not biased by the marginal sum of interaction frequencies in the matrix (i.e., *m* in equation 3; Figure S3c), which is the case for an existing specialization index that integrates availability and phylogenetic structure (Jorge et al., 2017; Appendix S1).

The higher rates of Type II error that we observed at the plot level of the CDMs were also reported by Miller et al. (2017) for other indices. The simulation strategy that we implemented to test Type II error is expected to generate the clustered and overdispersed patterns at the scale of the entire simulated arena. Our CDMs are intended to be a representative sample of that arena. Therefore, calculating PSS at the plot level (i.e., single rows of the matrix) is equivalent to taking a much smaller sample of that arena, which explains why the power of the index decreases. Therefore, we expect that the power of PSS will also decrease when interaction networks are under‐sampled, as is the case with other metrics (Blüthgen et al., 2008; Miller et al., 2017; Rivera‐Hutinel et al., 2012).

We urge caution when interpreting PSS for species with a single partner, because apparent specialization can be caused by the rareness of a species and not necessarily high phylogenetic specialization (e.g., Dorado et al., 2011). Our approach allows the calculation of PSS for species with a single partner, but in a way that will bias towards clustering when sampling is scarce. However, this is the case for all existing methods because true specialization can only be uncovered in the absence of artefacts such as imbalanced sampling effort (Blüthgen et al., 2008).

### 4.2 Phylogenetic structure in interaction networks

The integration of phylogenetic data with interaction networks can provide insights about the relative importance of ecological and evolutionary processes that shape biological communities (Segar et al., 2020). Previous studies have shown that many ecological interactions, as well as interaction‐related traits, display phylogenetic structure, where closely related species tend to have overlapping sets of partners (Aizen et al., 2016; Eklöf et al., 2012; Gómez et al., 2010; Rezende et al., 2007). Based on those findings, it should be common for species to be specialized on phylogenetically clustered partners. However, PSS analyses of four empirical networks showed that many species interact with phylogenetically random partners (Table 2; Figure 4d,h,l,p). Our results suggest that while interaction traits can be conserved across some phylogenetic scales, the assemblage of communities of interacting species at regional and local scales can be constrained by the relative effect of processes other than the evolutionary history of the species (Mello et al., 2019; Segar et al., 2020), such as the availability of potential partners.

Nevertheless, we also encountered many cases of phylogenetic specialization in all four empirical datasets (Table 2; Figure 4d,h,l,p). For example, in cyanolichens, the peak of the distribution of interactions was found to be in the random–clustered and clustered–clustered regions of the PSS space (Figures 2f and 4l). These results are consistent with past assessments that *Peltigera* species are most often specialized on generalist, but also on specialist, *Nostoc* phylogroups (Magain, Miadlikowska, Goffinet, et al., 2017). Similarly, Krasnov et al. (2012) reported that the fleas in the mammals–fleas dataset showed phylogenetic signal in their host range, which is consistent with our observed distribution of fleas infecting a clustered set of mammal hosts at a regional scale (Figures 2f and 4d). In addition, we detected mammal species that are infected by phylogenetically overdispersed fleas (Figures 2f and 4d). This pattern of overdispersion has not previously been reported for this dataset (Krasnov et al., 2012).

In contrast, the tropical avian seed‐dispersal network consists mostly of interactions involving generalist species (Figures 2d and 4f) that may not require specialized traits, or may be specialized on partner traits that are not phylogenetically conserved (Bello et al., 2017; Bolmgren & Eriksson, 2005; Emer et al., 2019). This dataset includes a large proportion (75%) of interactions involving species that associate with phylogenetically random partners (Figure 4h). However, the seed‐dispersal network also includes the largest proportion (22%) and most striking examples of interactions between species with clustered partners (Figures 2f and 4h). In the case of the hummingbird pollination dataset, we also found that most species interact with phylogenetically random partners (Table 2, Figure 4p). However, a previous study had already shown that more than half of the plant and hummingbird species in this network tend to interact with partners with morphologically matching traits (i.e., bill length and flower corolla length; Sonne et al., 2020). This may indicate that these traits are not phylogenetically conserved.

### 4.3 Is overdispersion a signature of specialists or generalists?

Studies that have used phylogenetic diversity metrics to characterize biotic specialization have often focused on cases where partners were significantly more closely related than expected by chance (but see Maherali & Klironomos, 2007) and considered overdispersion as a signature of generalists (Cooper et al., 2012; Jorge et al., 2014; Poulin et al., 2011). This is because overdispersion indicates that a species associates with distantly related partners. However, in a framework where partner availability is accounted for, a significant phylogenetic structure can only be detected when interaction frequencies are non‐random. With PSS, overdispersion means that a species interacts with its partners more than expected by chance, and those partners are more distantly related than expected by chance. This is consistent with high intensity of partner use within a narrow span of a species’ biotic niche and, therefore, should be interpreted as a signature of specialists (Figure 2c,f).

### 4.4 Availability based on interaction frequencies as a proxy for relative abundance in nature

Interaction frequencies in network matrices are commonly used as proxies for partner availability in nature, as evidenced by the widespread use of Blüthgen’s *d’* and related metrics to quantify specialization (Arceo‐Gómez et al., 2020; Fründ et al., 2016; Schleuning et al., 2012; Zanata et al., 2017). However, this proxy might be inaccurate if the interactions are not sampled systematically, when facultative partners are involved, or when interaction frequencies are independent from the availability of partners in nature (e.g., empirically shown in Vizentin‐Bugoni et al., 2014). We had direct empirical estimates of partner availability for the fleas from the mammals–fleas dataset and both the hummingbirds and plants in the hummingbird pollination dataset (Table 1; Sonne et al., 2020; Stanko et al., 2002). For the fleas and the plants, we found high correspondence among PSS values calculated based on empirically estimated availability and using interaction frequencies as a proxy for availability, but not for the hummingbirds (Figure 5). The availability proxy using interaction frequencies might be especially problematic for the *Peltigera–Nostoc* dataset, which was sampled at a global scale in a non‐systematic way. In this case, the interaction frequencies may lead to highly inaccurate estimates of the partner availabilities, particularly since *Nostoc* symbionts can be free‐living (Nelson et al., 2021).

### 4.5 Importance of phylogenetic and spatial scales for interpreting PSS values

Interpretations of PSS values must consider the phylogenetic and spatial scales of the datasets. For example, we found that a large proportion (54%) of cyanobacterial taxa associate with random partners (Table 2). However, this network only includes the interactions with species from a single genus of lichen‐forming fungi (*Peltigera*). If we had done the same analysis in the context of all lichen‐forming fungi (which span multiple classes of Fungi), the partners of many cyanobacterial taxa would be highly clustered and some would be overdispersed. Likewise, the avian seed‐dispersal dataset consists of interactions that were sampled in a single region, the Atlantic Forest of Brazil (Bello et al., 2017). Using PSS, we found that 39% of the interactions in this dataset involve plants whose seeds are dispersed by phylogenetically random birds (Table 2, Figure 4h). These sets of bird seed dispersers are phylogenetically random relative to the pool of species in the Atlantic Forest, but they likely represent a non‐random subset of the phylogenetic diversity of bird species at larger spatial scales, as shown by a continental‐scale study in South America (Mello et al., 2019).

### 4.6 A conceptual framework for an eco‐evolutionary interpretation of PSS values

Patterns of phylogenetic diversity are not direct proxies for community assembly processes (Cahill et al., 2008; Gerhold et al., 2015; Mayfield & Levine, 2010). Instead, we propose testable hypotheses of eco‐evolutionary processes that may produce PSS patterns in interaction networks.

Opportunistic interactions can result from multiple processes. Recent colonization or introduction (e.g., long‐distance dispersal events or invasive species) into new areas might make opportunistic interactions advantageous in ecological and evolutionary timescales (Magain, Miadlikowska, Goffinet, et al., 2017; Poisot et al., 2011). During rapid diversifications, incomplete sorting of traits can generate local populations with high intraspecific variation in interaction traits that allow associations with a broader range of partners. Species may also have spatially structured populations with low phenotypic variation at local scales, but higher variation at larger scales (Batstone et al., 2018). This highlights the importance of studying these patterns at multiple spatial scales (Gomulkiewicz et al., 2000; Jorge et al., 2014). Low heterogeneity in resources exchanged by partners can result in opportunistic interactions (Pinheiro et al., 2019). A recent study also showed that high ecological uncertainty can favor generalized host ranges in avian brood parasites (Antonson et al., 2020). How and when selection maintains the variation necessary for opportunistic interactions is not fully understood (Vamosi et al., 2014; but see Batstone et al., 2018), but it seems to be pervasive even in highly intimate symbioses such as lichens (Figure 4l; Guimarães et al., 2007).

Clustered patterns of biotic specificity may arise when the diversification dynamics of one set of organisms is dependent on its interacting partners. In rare cases, this may lead to cospeciation (de Vienne et al., 2013). More commonly, clustering results from repeated switches to closely related partners through time (Chagnon et al., 2019; Thines, 2019; de Vienne et al., 2013) or from the acquisition of a novel partner that promotes speciation of the interacting species, where emerging new species all retain compatibility with the novel partner (Chagnon et al., 2019; Gomulkiewicz et al., 2000).

Overdispersed patterns of phylogenetic specificity may arise through retention of plesiomorphic traits, convergent evolution, or competitive exclusion of related partners. Coevolutionary theory predicts that convergent evolution of interaction traits is common in mutualistic networks due to indirect selection pressures that spread throughout the networks (Guimarães et al., 2011, 2017). However, convergent evolution in interaction networks can also result in random phylogenetic structure if partner compatibility does not systematically evolve on closely or distantly related lineages.

## 5 CONCLUSION

Our approach presents a quantitative and conceptual framework to study specialization, and the eco‐evolutionary processes that shape it, in interaction networks. Importantly, the calculation of our PSS index allows the quantification of biotic specialization while accounting for partner availability and yielding values that are comparable across systems regardless of network properties. Furthermore, our PSS index can be used to elucidate the relationship between phylogenetic specialization and the distribution, abundance, and fitness of species in natural communities (Blüthgen et al., 2007; Fortuna et al., 2020; Pinheiro et al., 2016, 2019; Schleuning et al., 2012). This may have important implications for managing biodiversity when considering species interactions (Harvey et al., 2017).

## CONFLICT OF INTEREST

The authors declare no competing interests.

## AUTHOR CONTRIBUTIONS

**Carlos J. Pardo‐De la Hoz:** Conceptualization (equal); Data curation (lead); Formal analysis (lead); Funding acquisition (equal); Methodology (lead); Software (lead); Writing – original draft (lead); Writing – review & editing (equal). **Ian D. Medeiros:** Conceptualization (equal); Methodology (supporting); Visualization (supporting); Writing – review & editing (equal). **Jean Philippe Gibert:** Conceptualization (supporting); Methodology (supporting); Writing – review & editing (supporting). **Pierre‐Luc Chagnon:** Conceptualization (supporting); Methodology (supporting); Resources (equal); Writing – review & editing (supporting). **Nicolas Magain:** Resources (equal); Writing – review & editing (supporting). **Jolanta Miadlikowska:** Funding acquisition (equal); Resources (equal); Writing – review & editing (supporting). **François Lutzoni:** Conceptualization (equal); Funding acquisition (equal); Methodology (supporting); Project administration (lead); Supervision (lead); Writing – original draft (supporting); Writing – review & editing (equal).

## Supporting information

Appendix S1‐S3

## Data Availability

The PSS R package is available at https://github.com/cjpardodelahoz/pss. All datasets, including empirical and simulated interaction matrices and trees, as well as all the code used in this study, are available from the Dryad Digital Repository: https://doi.org/10.5061/dryad.s1rn8pk4q.
